# Fetal Rhabdomyoma of the Right Tonsil with Polyp-Like Appearance

**DOI:** 10.1155/2015/713278

**Published:** 2015-07-13

**Authors:** Ching-Ping Wang, Yi-Hao Chang, Ya-Ting Chang

**Affiliations:** ^1^Department of Otolaryngology, Taichung Veterans General Hospital, 1650 Taiwan Boulevard Section 4, Taichung 40705, Taiwan; ^2^Department of Stomatology, Taichung Veterans General Hospital, 1650 Taiwan Boulevard Section 4, Taichung 40705, Taiwan

## Abstract

Skeletal muscle neoplasms, in contrast to other groups of tumors, are almost malignant. The benign variant, rhabdomyoma, is distinctly rare. Rhabdomyomas can be classified generally into two types: cardiac and extracardiac. Extracardiac rhabdomyoma can be further divided into three subtypes: adult, fetal, and genital type. Adult rhabdomyoma is the most common subtype of rhabdomyoma even though it remains relatively rare. Fetal rhabdomyomas are less common than the adult type. In this paper we report a rare case of a fetal rhabdomyoma with polyp-like appearance originating from right tonsil. Punch biopsy and then right tonsillectomy were performed for complete excision. There was no obvious recurrence.

## 1. Introduction

The term “rhabdomyoma” was introduced by Zenker in 1864. It was used to describe a benign tumor showing muscle cells with different degrees of differentiation and maturity. Rhabdomyomas are rare benign tumor consisting of striated muscle. They can be classified generally into two types: cardiac and extracardiac [1]. Most rhabdomyomas arise from cardiac muscle. Cardiac rhabdomyoma occurs almost exclusively in the pediatric age group and may be associated with tuberous sclerosis, neurofibromatosis, and sebaceous adenomas [2].

Extracardiac rhabdomyoma can be further divided into three subtypes (adult, fetal, and genital) with regard to clinical and morphological differences, although some overlapping exists. Genital rhabdomyoma is seen most frequently in the vagina or vulva of young and middle-aged women. Fetal and adult rhabdomyoma are located predominantly in the head and neck region; the former occurs primarily in the subcutaneous tissue of the head and neck of male infants usually younger than 3 years, and the latter is characterized by a slowly growing mass typically seen in the head and neck of elderly patient, with a male predominance. Seventy-seven percent of all extracardiac rhabdomyomas occur in the head and neck and 14% occur in the genital region [3]. Adult rhabdomyoma is the most common subtype of rhabdomyoma even though it remains relatively rare. Fetal rhabdomyomas are less common than the adult type. In this paper we report a rare case of a fetal rhabdomyoma with polyp-like appearance originating from right tonsil.

## 2. Case report

**Figure 1:**
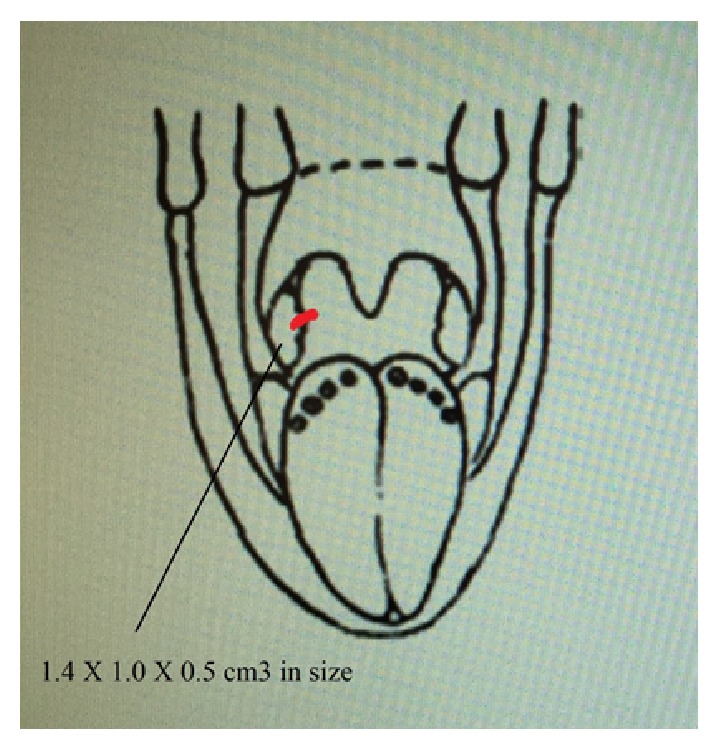
A polyp over right tonsil.

**Figure 2:**
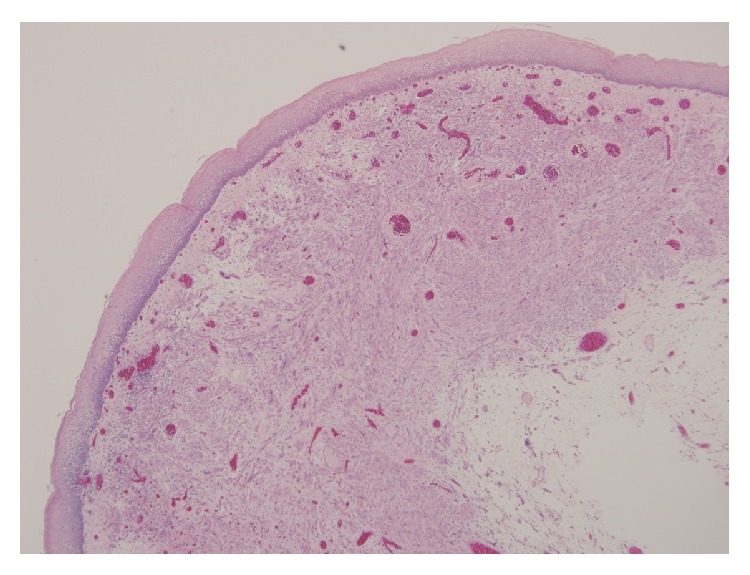
Haphazardly arranged muscle fibers under squamous mucosa (hematoxylin and eosin stain, 40x).

**Figure 3:**
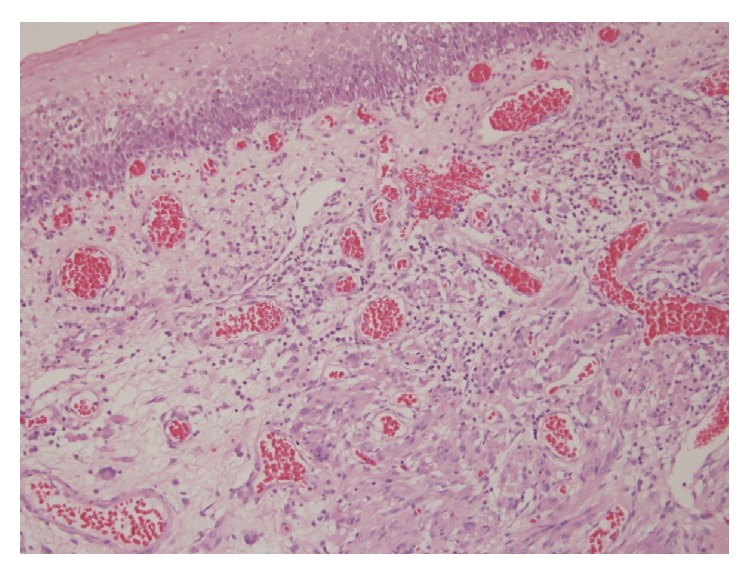
Skeletal muscle fibers found in submucosa of tonsil with vascular proliferation (hematoxylin and eosin stain, 100x).

**Figure 4:**
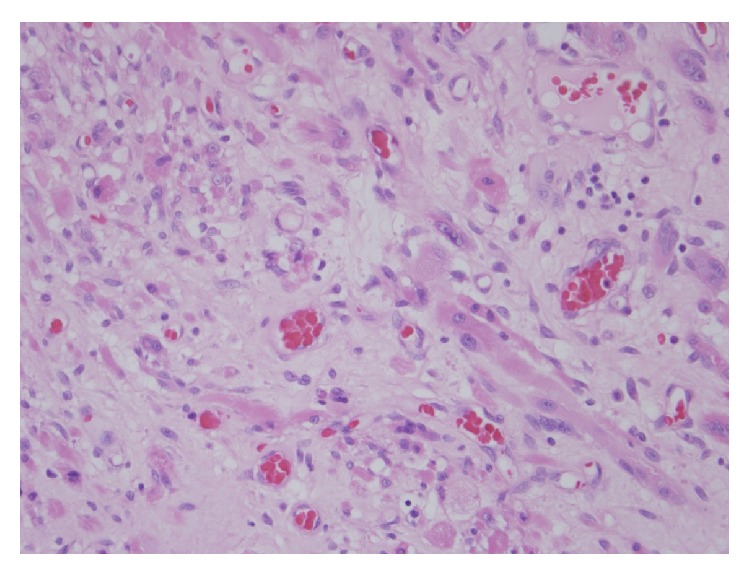
Muscle fibers in loose and myxoid stroma. Note the elongated cells with eosinophilic cytoplasm (hematoxylin and eosin stain, 200x).

**Figure 5:**
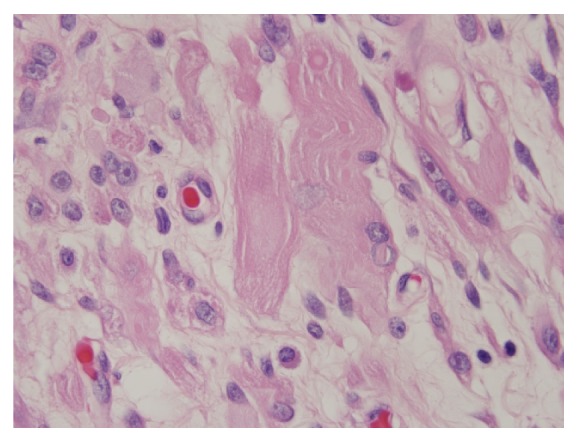
Skeletal muscle differentiation with striation is noted (hematoxylin and eosin stain, 400x).

**Figure 6:**
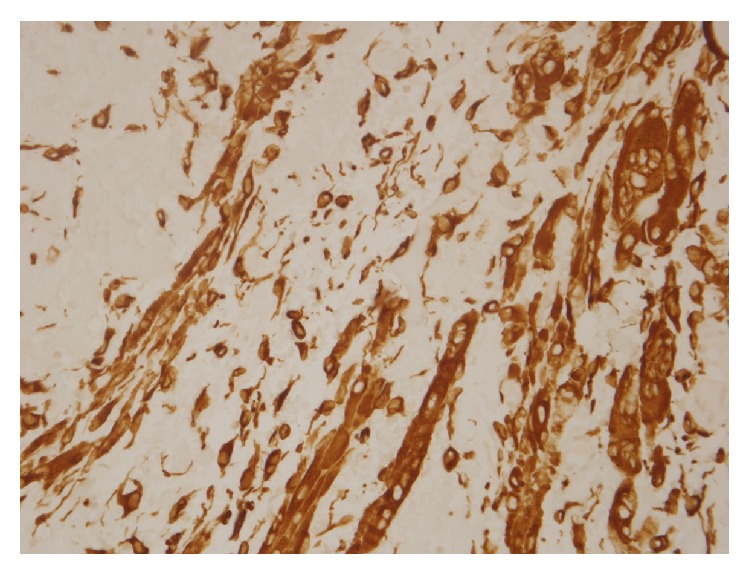
IHC stain for desmin is positive (hematoxylin and eosin stain, ×400).

**Figure 7:**
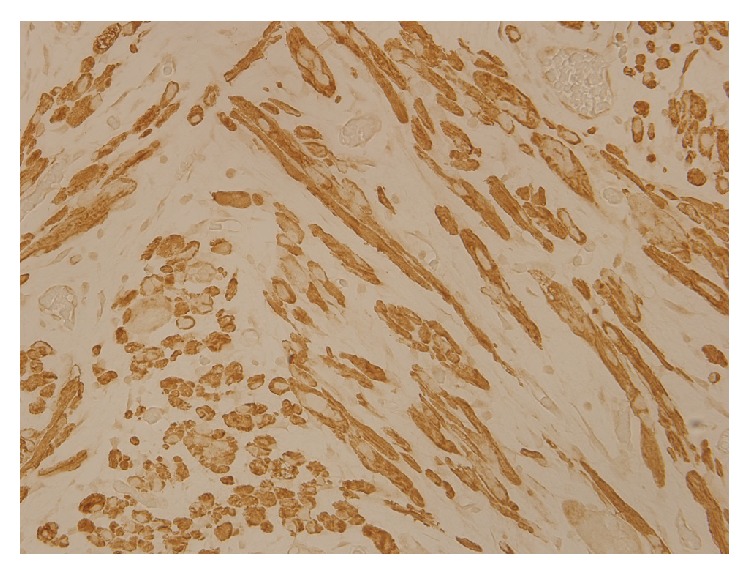
IHC stain for actin M874 is positive (hematoxylin and eosin stain, ×400).

A 57-year-old patient presented to our otolaryngology department on August 17, 2009, with a small painless polyp which he has had over right tonsil for one month. This patient had past history of diabetes mellitus under regular oral hypoglycemic agents control for more than 5 years. He denied any other medical or surgical history. Physical examination revealed a smooth surface polyp about 1.4 × 1.0 × 0.5 cm^3^ over right tonsil without bleeding or ulceration (Figure 1); there was no other oral lesion or palpable neck lymph node. Fiberoptic nasolaryngoscopy was performed and the finding was unremarkable. We thought the polyp was a benign lesion, so we did not pay much attention to it and did not take a photo for documentation. We performed punch biopsy under local anesthesia. Then histopathological analysis showed polypoid squamous epithelial lining of the lesion. The submucosa showed interlacing cellular spindle and epithelioid rhabdomyomatous tumor cell proliferation with degenerative cellular atypia. There were haphazardly arranged muscle fibers under squamous mucosa. Skeletal muscle fibers with vascular proliferation were found in submucosa of tonsil (Figures 2 and 3). We also observed muscle fibers in loose and myxoid stroma. The cells were elongated and had eosinophilic cytoplasm. Skeletal muscle differentiation with striation was noted (Figures 4 and 5). Striation is an evidence of skeletal muscle. Testing for immunohistochemical markers desmin is positive (Figure 6). IHC for actin M874 was also done and positive finding was noted (Figure 7). The differential diagnosis of rhabdomyoma and rhabdomyosarcoma depends on the degree of maturation. In this specimen, there are no small blue round cells, and all of the neoplastic cells show mature skeletal muscle features. That means uniform population of differentiating myoblasts. Besides, in this specimen, no nuclear atypia, atypical mitotic figures nor necrosis were found. Rhabdomyosarcoma is primitive neoplasm with predominantly small blue round cells.

Deep resection margin could not be completely evaluated due to fragmentation of specimen. Final diagnosis was fetal rhabdomyoma. Surgical excision is the imperative treatment for rhabdomyoma, so we arranged right tonsillectomy under general anesthesia on August 31, 2009. Pathologic report showed focal granulation with chronic inflammation and reactive lymphoid hyperplasia. There was no rhabdomyoma noted on this specimen. The result indicated that polyp-appearance rhabdomyoma was excised completely when we performed punch biopsy and there was no residual rhabdomyoma on the tonsil. After operation, the patient did not come back to our department for follow-up. We contacted this patient by phone and he said he had no discomfort or abnormal lesion over right oropharynx. There was no obvious recurrence.

## 3. Discussion

Rhabdomyoma is an exceedingly rare soft tissue benign tumor derived from skeletal muscle. The pathogenesis is unclear. As a general rule, benign soft tissue tumors occur more frequently than their malignant counterparts, but this does not hold true for striated muscle tumors because rhabdomyomas account for only 2% of skeletal muscle tumors [4]. Fetal rhabdomyoma was first differentiated from adult rhabdomyoma by Dehner et al. based on the presence of elongated immature rhabdomyocytes in varying stages of differentiation [5]. Fetal rhabdomyomas are less common than the adult type. The fetal type occurs primarily in the subcutaneous tissue of the head and neck of male infants younger than 3 years old. In this presented case, the onset age, location, and polyp-like appearance were all unusual. In the literature, no other case of fetal rhabdomyoma over tonsil has been reported.

Regarding therapeutic options for this tumor, the gold standard of treatment is surgery. Rhabdomyoma may recur, but no cases of malignant transformation have been described in the literature. Recurrence is probably due to incomplete excision or multifocality. When total excision cannot be accomplished, reoperation or narrow follow-up is indicated to prevent advanced revision surgeries. In this presented case, complete excision was done, and there was no obvious recurrence.

## 4. Conclusion

Skeletal muscle neoplasms, in contrast to other groups of tumors, are almost always malignant. The benign variant, rhabdomyoma, is distinctly rare. Our patient was a rare case of fetal rhabdomyoma located in right tonsil. Initially we thought it was a polyp over right tonsil and performed punch biopsy. Pathology report showed fetal rhabdomyoma, and curative treatment was obtained by surgery. There was no obvious recurrence.

Although rhabdomyoma of tonsil is a rare disease, we should keep in mind this differential diagnosis when we meet patient with polyp over tonsil or other H&N areas. Be sure to take photodocumentation routinely before punch biopsy or excision even though it looks like benign lesion. If you meet patient with rhabdomyoma in the future, surgical excision is an imperative treatment. Recurrence rate is extremely low when complete excision is done.
